# Validation of the Measurement of Beta-Hydroxybutyrate and Non-Esterified Fatty Acids in Bovine Saliva: A Pilot Report

**DOI:** 10.3390/life15060854

**Published:** 2025-05-26

**Authors:** Camila P. Rubio, Lucas Rigueira, Marta Miranda, Pedro Javier Vallejo, Jesús Semitiel, David del Olmo, María D. Contreras-Aguilar, Flávio G. Silva, Elsa Lamy, Christian De la Fe, José J. Cerón, Fernando Tecles

**Affiliations:** 1 Interdisciplinary Laboratory of Clinical Analysis (Interlab-UMU), Veterinary School, Regional Campus of International Excellence ‘Campus Mare Nostrum’, University of Murcia, Campus de Espinardo, Espinardo, 30100 Murcia, Spain; camila.peres@um.es (C.P.R.); pedrojavier.vallejo@um.es (P.J.V.); mariadolores.contreras@um.es (M.D.C.-A.); jjceron@um.es (J.J.C.); 2 Departamento de Anatomía, Producción Animal y Ciencias Clínicas Veterinarias, Servicio de Animales de Renta, Hospital Veterinario Universitario Rof-Codina, Facultad de Veterinaria, Campus Terra, Universidad de Santiago de Compostela, 27002 Lugo, Spain; lucas.rigueira@usc.es (L.R.); marta.miranda@usc.es (M.M.); 3 JISAP, 30817 Lorca, Spain; jsemitiel@jisap.com (J.S.); davidolmo@jisap.com (D.d.O.); 4 MED-Mediterranean Institute for Agriculture, Environment and Development, University of Évora, Pólo da Mitra, Ap. 94, 7006-554 Évora, Portugal; fsilva@uevora.pt (F.G.S.); ecsl@uevora.pt (E.L.); 5 CHANGE—Global Change and Sustainability Institute, University of Évora, Pólo da Mitra, Ap. 94, 7006-554 Évora, Portugal; 6 Ruminant Health Research Group, Department of Animal Health, Faculty of Veterinary Sciences, Regional Campus of International Excellence “Campus Mare Nostrum”, University of Murcia, 30100 Murcia, Spain; cdelafe@um.es

**Keywords:** biomarkers, bovine, energy balance, ketone bodies, saliva

## Abstract

Serum beta-hydroxybutyrate (BHB) and non-esterified fatty acids (NEFAs) are biomarkers of situations of negative energetic balance in bovine. However, knowledge about their possible measurement and use in saliva is limited. In this report, two commercially available methods for the measurement of BHB and NEFAs were validated for use in bovine saliva. Both methods showed good precision and accuracy. The BHB concentrations were correlated between the saliva and the serum, but not the NEFA concentrations. The cows with hyperketonemia (*n* = 17) had increased salivary BHB compared to the cows with no clinical signs and no hyperketonemia (*n* = 34) and those with clinical signs of metritis (*n* = 17). The salivary NEFA concentration increased in newborn calves (*n* = 10) on days 1 and 2 of life compared to the day of birth before colostrum intake. The calves with symptomatic bovine respiratory disease complex (BRD, *n* = 7) showed higher salivary NEFA concentrations than those without clinical symptoms (*n* = 6). Thus, BHB and NEFAs can be reliably measured in bovine saliva using easily automatable colorimetric methods. Salivary BHB increased in hyperketonemia and could be a potential biomarker of this condition. Further studies should be undertaken to clarify the mechanism and possible use of salivary NEFAs as biomarkers.

## 1. Introduction

Hyperketonemia is defined as an increase in the blood concentrations of ketone bodies. This is a common metabolic disorder in dairy cows, especially in the Holstein breed, and its prevalence could reach around 22.7% in herds [1]. Pathogenesis is related to the increase in glucose and amino acid requirements at peripartum and lactation, physiological states in which cows experience an important increase in energy demands [2,3]; often accompanied by a reduction of dry matter intake [4], leading to a negative energy and nitrogen balance. This situation can last from 3 weeks before calving until 3 weeks after, due to the high energy requirements derived from fetal growth and milk synthesis, among other factors [5,6]. In order to face this challenge, cows mobilize body reserves such as lipids and protein [7]. Ketone bodies such as beta-hydroxybutyrate (BHB) appear in the liver as a result of the partial oxidation of non-esterified fatty acids (NEFAs), which are mobilized in those situations of increased energy requirements [8,9]. Serum levels of BHB are used for the diagnosis, with values higher than 1.2–1.4 mmol/L being a positive result in the bovine species. Values between 1.2 and 3.0 mmol/L are called hyperketonemia and are associated with a state of subclinical ketosis in which the clinical signs are less apparent but fertility could be affected [10]. In addition, hyperketonemia has been related to the presence of other disorders at peripartum such as displaced abomasum, mastitis, metritis, lameness, fatty liver, reproduction issues, and a greater risk of culling [11]. Clinical ketosis usually appears when BHB levels are over 3.0 mmol/L and includes neurological issues, among others [10,12]. Both clinical and subclinical ketosis suppose important economic losses, and therefore, prevention and early detection followed by adequate treatment are crucial [13].

Increases in NEFAs can also be related to other situations. For example, they have been reported to be increased in calves in the first days of life, reaching values similar to those found in adults by the second week [14]. NEFAs are also increased in cows with metritis [15], a prevalent peripartum disease that occurs shortly after calving due to a bacterial infection producing vaginal discharge [16,17]. This could be produced by the ability of NEFAs to affect neutrophils’ function at elevated concentrations [18], reducing their oxidative burst function [19]. Despite the existing data about hyperketonemia and the increase in NEFAs in some diseases, there remain other diseases where, to the authors` knowledge, these analytes have not been studied. An example is bovine respiratory disease complex (BRD), a multifactorial and polymicrobial complex that primarily involves both bacteria and viruses, in addition to environmental components, host, and management factors [20].

As an analytical sample, saliva has several advantages over blood, since it can be easily collected with no need for specific personnel training. In addition, it can be obtained safely without producing any stress or damage to the animals, thus allowing repeated sampling with no disturbance even when a high sampling frequency is required [21]. Cow saliva contains several biomarkers that can be successfully analyzed, including stress, inflammatory, oxidative, and general metabolism biomarkers [22,23]. However, to the authors’ knowledge, saliva has not yet been used either for the measurement of BHB or NEFAs in bovine species. Thus, the main aim of this study was to evaluate whether BHB and NEFAs could be measured in saliva in bovine. For this purpose, two commercial colorimetric methods for the measurement of BHB and NEFAs were validated. In addition, in order to obtain information about possible changes to these analytes during physiological conditions and disease, the salivary concentrations of the biomarkers were measured in adult cows with hyperketonemia or metritis, in calves before and after colostrum intake, and in calves with symptomatic BRD.

## 2. Materials and Methods

### 2.1. Animals and Sampling

A total of 73 adult Holstein Friesian cows at the peripartum period and 23 calves of different ages were included in this study. The characteristics of the animals are shown in Section 2.4 (Clinical validation).

Saliva was collected from animals by introducing a small sponge (3 × 3 × 5 cm) clipped to a flexible thin metal rod into the mouth. The animals were free to chew the sponge until it was thoroughly moistened. Then, the sponge was introduced into a tube (Salivette, Sarstedt, Aktiengesellschaft & Co, Nümbrecht, Germany). After saliva sampling, blood was obtained by venipuncture (jugular vein in calves and coccygeal vein in cows) using a plain tube. Saliva and blood samples were stored on ice until arrival at the laboratory in less than 2 h. Then, both saliva and blood tubes were centrifuged (3.000× *g* for 10 min at 4 °C) and supernatants collected. Saliva and serum samples were stored at −80 °C until analysis. Verbal informed consent was obtained from all farm owners for sampling.

### 2.2. Analytical Methods

Specific commercial kits were used. For the determination of NEFAs (Ref. FA115; RANDOX Laboratories, Life Sciences Ltd., Crumlin, UK), those present in the sample react with CoA in presence of Acyl CoA synthetase to produce Acyl CoA that is then oxidized to produce hydrogen peroxide (H_2_O_2_). The amount of H_2_O_2_ is quantified, being proportional to the amount of NEFAs present in the sample. The method for BHB measurement (RB1007, RANDOX Laboratories Ltd., Crumlin, UK) is based in the oxidation of BHB to acetoacetate by the enzyme hydroxybutyrate dehydrogenase. At the same time, the cofactor NAD^+^ is reduced to NADH. Analyses were carried out on an automated chemistry analyzer (Olympus Diagnostica GmbH AU 400, Beckman Coulter, Ennis, Ireland) following the assay manufacturer’s instructions. The specific calibrators provided in the commercial kits were used to calibrate each method.

### 2.3. Analytical Validation

Analytical performance of the assays was assessed through the following calculations:

(a). Imprecision. It was assessed by calculating the coefficient of variation (CV) from a batch of determinations. Two saliva samples, one with a high and another with a low concentration of the analyte, were analyzed six times in the same analytical batch for intra-assay CV calculation. For inter-assay CV, the two samples with different concentrations were analyzed six times on different days. Samples were aliquoted and stored frozen at −80 °C; a different aliquot was used each day in order to avoid any effect due to freezing–thawing cycles. CVs were calculated as $\frac{\mathrm{Standard deviation}}{\mathrm{mean}}$ × 100.
(b). Accuracy. It was indirectly estimated by linearity under dilution assays. Two samples of known analyte concentration were serially diluted with ultrapure water and then analyzed. Regression plots were constructed by facing observed vs. expected results in order to check whether slope was statistically different from zero.
(c). Lower limit of detection (LLOD). It is defined as the lowest concentration capable of being detected by the assay. For its calculation, the assay diluent (ultrapure water) was analyzed 20 times in the same analytical batch. Then, the LLOD was estimated as mean+(3×standard deviation).
(d). Influence of dirtiness in the saliva sample. Saliva samples from the 73 adult cows were visually inspected and given a score depending on the presence of color and dirt in the sample, with a value of 0 being given when the sample showed no color or dirt and a value of 4 when the sample was cloudy, colored, and showed the presence of sediment, according to a previously published protocol [24]. Then, regression studies were performed in order to evaluate the effect of dirtiness on the assay results. In case of a significant effect, the most colored samples (scoring with 4) would be excluded for the rest of the assays.
(e). Correlation between values in saliva vs. serum. In order to evaluate the possible correlations between salivary and serum levels, regression plots were constructed comparing the values of the different biomarkers obtained from paired saliva and serum samples from adult cows.

### 2.4. Changes in Physiological Conditions and Disease

For the clinical validation of the assays, different approaches were performed in adult cows and in calves.

#### 2.4.1. Changes in Salivary Biomarkers in Adult Cows with Hyperketonemia or Metritis

Saliva and serum samples were collected from adult cows stabled in one conventional intensive dairy farm located in Lugo (Spain). Cows were fed a standardized total mixed ration (TMR) formulated for high-producing groups. All animals were Holstein Friesian cows with a standardized mature body weight of approximately 680 kg, a similar health status, and an average 305-day corrected milk yield of around 13,000 kg. All samples were collected before the first meal of the day. Three groups of animals were considered: cows with neither clinical signs nor hyperketonemia (serum BHB < 1.2 mmol/L), cows without clinicals signs but hyperketonemia (serum BHB > 1.2 mmol/L), and cows with clinical signs of metritis without hyperketonemia. The characteristics of each group are described below.

(a). The group of animals with neither clinical signs nor hyperketonemia (NCS-NH) was composed of 38 Holstein Friesian cows with a median age of 4.00 (Interquartile Range = 1.0) years old, median body condition score (BCS) of 2.9 (IQR = 0.3), median parity of 3.0 (IQR = 3.0), and median 26.0 (IQR = 59.5) days in milk (DIM). The animals did not show any evidence or sign of clinical disease at the time of sampling.
(b). The cows without clinical signs but hyperketonemia (NCS-H) included 17 Holstein Friesian animals with a median age of 5.0 (IQR = 5.0) years old and a median BCS of 3.0 (IQR = 0.9). They had a median parity of 3.0 (IQR = 2.0) and a median of 14.3 (IQR = 8.1) DIM. The animals had no clinical signs but were all diagnosed with hyperketonemia since all of them had serum BHB levels > 1.2 mmol/L [12] using a GlucoMen LX β-Ketone Sensor (A. Menarini GmbH, Zürich, Switzerland), a method validated for bovine species [25].
(c). The cows with clinical signs of metritis without hyperketonemia (CSM-NH) included 18 Holstein Friesian animals with a median age of 4.0 (IQR = 2.0) years old and median BCS of 2.8 (IQR = 0.6). They had a median parity of 3.0 (IQR = 3.0) and a median of 14.0 (IQR = 11.0) DIM. The diagnosis was based on visual inspection and clinical examination, according to previous research [26]. In brief, animals showed clinical signs compatible with metritis (enlarged uterus and fetid abnormal uterine discharge, associated with signs of systemic illness) within 21 days after parturition, and other potential complications such as lameness or ketosis were excluded.

#### 2.4.2. Changes in Salivary Biomarkers in Healthy Calves over Time

In this trial, serum and saliva samples were obtained from a group of 10 newborn Holstein Friesian calves from a commercial dairy farm located in Évora (Portugal). Samples were obtained the same day of calving 30 min before the colostrum intake (Day 0). Then, the animals were sampled again on days 1, 2, and 7 after birth. The calves’ health assessments were performed on each day of analysis with an adapted version of the Wisconsin calf health scoring system [27] and none of the animals showed any symptomatology of disease.

#### 2.4.3. Changes in Salivary Biomarkers in Calves with Bovine Respiratory Disease

In this experiment, samples were obtained from a farm in Murcia (Spain). A group of asymptomatic calves (N-BRD group, *n* = 6, mixed breed, 7–8 months old) and a second group of animals with clinical signs of BRD complex (BRD group, *n* = 7, mixed breed, 7–8 months old) were sampled. Depending on the case, the symptoms included ocular and nasal discharge, cough, extended head, dry snout, floppy ears, poor coat, dull eyes, lethargy, social isolation, hypo-/anorexia, pyrexia, dehydration, tachypnea, and dyspnea. At the time of sampling, none of the animals had received any vaccination. In both groups, the presence of infection for selected pathogens commonly involved in BRD was evaluated by polymerase chain reaction (PCR) assays. Briefly, samples were collected using a BOVIRESP^®^ kit (HIPRA, Gerona, Spain), designed to collect nasal exudate from calves. Refrigerated samples were sent to DIAGNOS (HIPRA). Once in the laboratory, automated extraction of DNA and RNA (QIAmp DNA and RNeasy minikit, Qiagen, Germany) was performed (QIAcube, Qiagen), and then specific amplification of genetic material was performed through three different RT-PCRs adapted from previously described protocols [28,29,30,31]. The asymptomatic calves included 33.3% of animals positive to bovine viral diarrhea virus (BVDV), 50% positive to Parainfluenza-3 (PI-3), 16.7% positive to bovine respiratory syncytial virus (BRSV), 100% positive to *Mannheimia haemolitica*, 50% positive to *Histophilus sommi*, 100% positive to *Mycoplasma bovis*, and 16.7% positive to *Pasteurella multocida*. The symptomatic group included 57.1% calves positive to BVDV, 14.3% positive to PI-3, 14.3% positive to BRSV, 100% positive to *M. haemolitica*, 28.6% positive to *H. sommi*, 100% positive to *M. bovis*, and 28.6% positive to *P. multocida*.

### 2.5. Statistical Analysis

All data were first assessed for normality through the Shapiro–Wilk normality test, resulting in non-parametric distribution. Linear regressions were performed to assess linearity under dilution study, the relationship between biomarker concentrations in saliva and the presence of dirtiness, and salivary vs. serum levels. The biomarker concentrations between the groups of cows (NCS-NH, NCS-H, and CSM-NH) were compared by Kruskal–Wallis’s test, followed by Dunn’s multiple comparison test. The suitability of the biomarkers for the diagnosis of hyperketonemia was assessed by receiver operating characteristic (ROC) analysis, and the area under the curve (AUC) was calculated. For this, two groups of animals were considered: (a) animals without hyperketonemia (NCS-NH and CSM-NH groups); and (b) the group of animals with hyperketonemia (NCS-H group). The changes that happened in serum and saliva of newborn calves during the first week of life were assessed by Friedman’s test, followed by Dunn’s multiple comparison test. Differences in serum and saliva analytes between asymptomatic calves and calves with symptomatic BRD complex were assessed by Mann–Whitney test. Eta^2^, Kendall’s W, and ***r*** coefficient were estimated for the calculation of effect size for Kruskal–Wallis, Friedman, and Mann–Whitney tests, respectively. The analyses were performed using the statistical package GraphPad Prism, version 9 for Mackintosh (Graph Pad Software Inc., San Diego, CA, USA). The significance was set at α = 0.05.

## 3. Results

### 3.1. Analytical Validation

**Figure 1:**
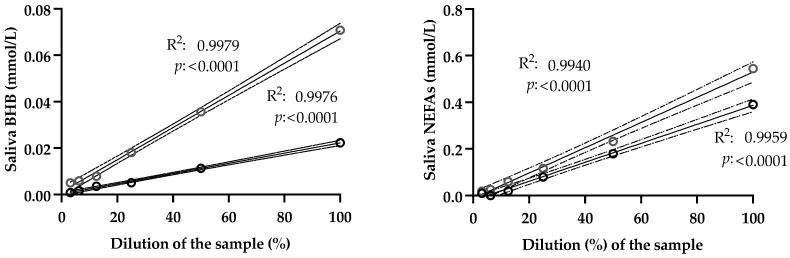
Linearity under dilution assays performed in saliva samples with high beta-hydroxybutyrate (BHB) and non-esterified fatty acids (NEFAs), serially diluted with distilled water. Dotted lines indicate the 95% confidence interval. R^2^: coefficient of determination.

The results of the imprecision study are shown in Table 1. The analytical methods provided intra- and inter-assay imprecisions of <3% and <6%, respectively, for samples with a high concentration of the analytes. When the concentration was low, intra- and inter-assay imprecisions were <7.1% and <14%, respectively. The results of the linearity under dilution assays are shown in Figure 1. The results showed regression coefficients R^2^ > 0.99. The LLOD was 0.005 mmol/L for BHB and 0.015 mmol/L for NEFAs.

**Figure 2:**
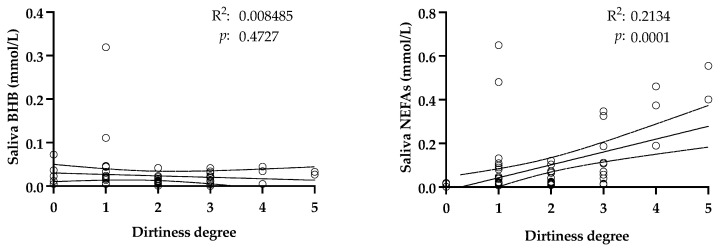
Regression plots between levels of beta-hydroxybutyrate (BHB) and non-esterified fatty acids (NEFAs) in saliva from 73 adult cows and the color score of the samples. Dotted lines indicate the 95% confidence interval. R^2^: coefficient of determination.

The influence of dirtiness in the sample is shown in Figure 2. The presence of color in the sample did not affect the BHB values in saliva. In contrast, the NEFA concentrations were positively affected by the color present in the sample. Based on these results, the samples with a high color score (4 or 5) were excluded from further assays in this report, that is, 5 of 73 samples in the study of the changes in hyperketonemia and metritis. After the exclusion of those values, no linear relationship was observed between the salivary BHB and NEFA concentrations and dirtiness, with R^2^ = 0.02 (*p* = 0.259) and R^2^ = 0.01 (*p* = 0.465), respectively.

**Figure 3:**
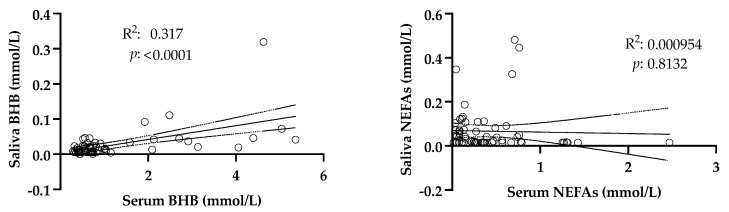
Regression plots constructed with the values of beta-hydroxybutyrate (BHB) and non-esterified fatty acids (NEFAs) obtained in paired serum–saliva samples from 68 adult cows. Dotted lines indicate the 95% confidence interval. R^2^: coefficient of determination.

The regression plots constructed between the salivary and serum levels of the analytes are shown in Figure 3. A significant linear relationship was found between the salivary and serum levels of BHB. The salivary NEFAs did not show a significant linear relationship with serum.

### 3.2. Changes in Adult Cows with Hyperketonemia or Metritis

**Figure 4:**
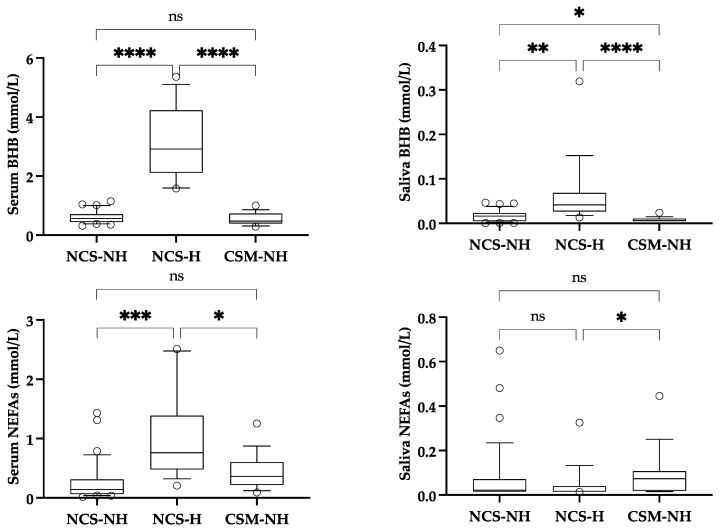
Concentrations of beta-hydroxybutyrate (BHB) and non-esterified fatty acids (NEFAs) in serum (**left**) and saliva (**right**) of cows with neither clinical signs nor hyperketonemia (NCS-NH), cows with no clinical signs but hyperketonemia (NCS-H), and cows with clinical signs of metritis without hyperketonemia (CSM-NH). Boxes show first and third quartiles, the line inside the box indicates median value, and whiskers indicate 10–90 percentiles. Statistical results: asterisks indicate significant differences between groups (*: *p* < 0.05; **: *p* < 0.01; ***: *p* < 0.001; ****: *p* < 0.0001); ns: non-significant. Eta^2^ was 0.54 for serum BHB, 0.40 for saliva BHB, 0.36 for serum NEFAs, and 0.05 for saliva NEFAs (from 0.01 to < 0.05: small effect; from 0.06 to <0.13: moderate effect; ≥0.14: large effect).

The results obtained in the different groups of cows are shown in Figure 4. After the exclusion of dirty samples, the NCS-NH, NCS-H, and CSM-H groups were composed of 34, 17, and 17 cows, respectively.

In the serum, BHB was significantly increased in the NCS-H group compared with the NCS-NH and CSM-NH groups. A similar finding was observed in the saliva, although the significance was lower between the NCS-NH and NCS-H groups, and the CSM-NH group also showed significant lower values than the NCS-NH group. In the serum, NEFAs were significantly increased in the animals in the NCS-H and CSM-NH groups compared with the animals in the NCS-NH group. No significant differences in saliva NEFAs were seen between groups.

**Figure 5:**
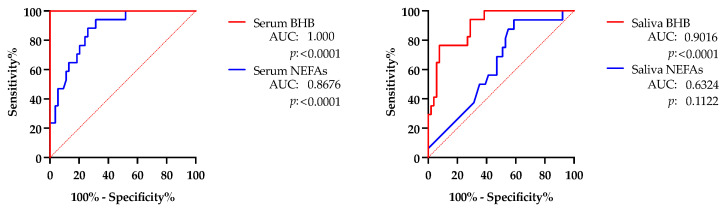
Receiver operating characteristic (ROC) curves and area under the curve (AUC) obtained with beta-hydroxybutyrate (BHB) and non-esterified fatty acids (NEFAs) in serum (**left**) and saliva (**right**) for discriminating cows with hyperketonemia (NCS-H group) from those without hyperketonemia (NCS-NH and CSM-NH groups).

The ROC assays (Figure 5) showed a better performance for detecting cows with hyperketonemia (NCS-H group) in the case of BHB and NEFAs in the serum, with an AUC =1.0 and >0.85, respectively. In the saliva, BHB was also able to significantly discriminate against hyperketonemic cows with a lower AUC than in the serum. The salivary NEFAs did not show usefulness for detecting cows with hyperketonemia.

### 3.3. Changes in Newborn Calves During the First Week of Life

**Figure 6:**
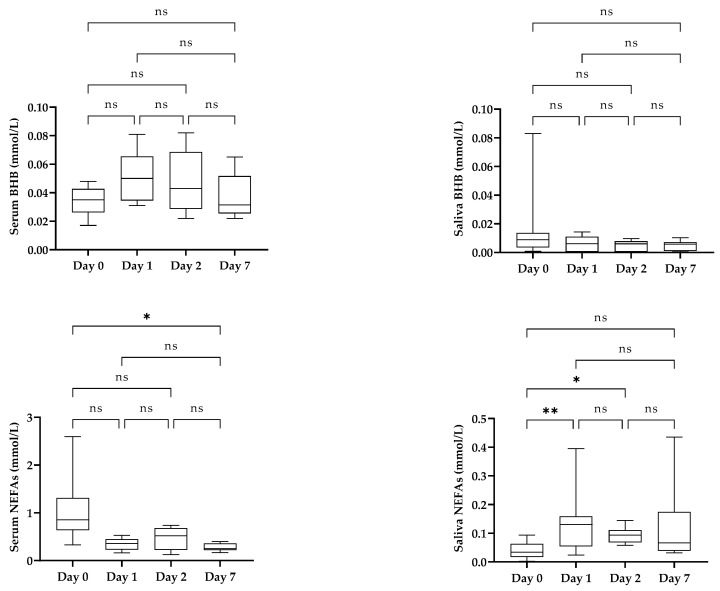
Evolution of beta-hydroxybutyrate (BHB) and non-esterified fatty acids (NEFAs) in serum and saliva of newborn calves (*n* = 10) during the first week of life. Boxes show first and third quartiles, the line inside the box indicates median value, and whiskers indicate 10–90 percentiles. Statistical results: asterisks indicate significant differences (*: *p* < 0.05; **: *p* < 0.01; ns: non-significant). Kendall’s W was 0.08 for serum BHB, 0.26 for saliva BHB, 0.32 for serum NEFAs, and 0.58 for saliva NEFAs (from 0.1 to <0.3: small effect; from 0.3 to <0.5: moderate effect; ≥0.5: large effect).

Figure 6 shows the serum and salivary concentrations of BHB and NEFAs in newborn calves from the day of birth until day 7 after birth. The BHB concentration did not show significant changes during the first week of life in either the serum or the saliva. Regarding NEFAs, no significant changes were observed in the serum despite the higher values that were observed on the day of calving. In contrast, in saliva, the NEFA concentration was significantly lower on day 0 compared to the rest of the days.

### 3.4. Calves with or Without Clinical Manifestations of BRD

**Figure 7:**
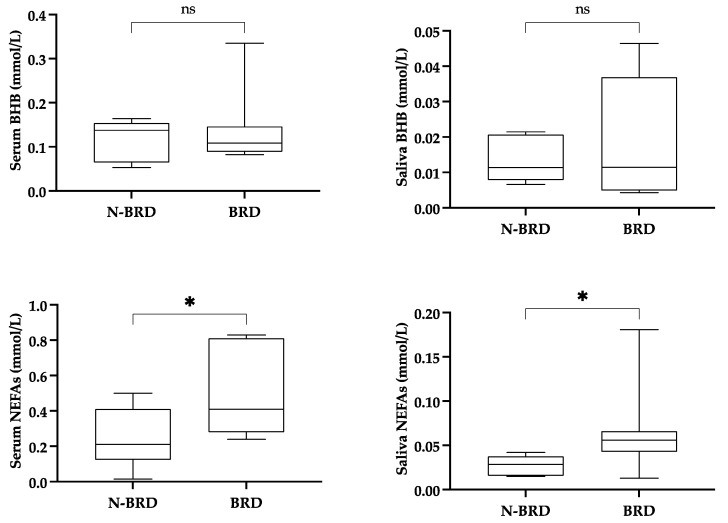
Levels of beta-hydroxybutyrate (BHB) and non-esterified fatty acids (NEFAs) in serum and saliva of calves with no clinical signs (N-BRD, *n* = 6) and calves with clinical signs of Bovine Respiratory Disease complex (BRD, *n* = 7). Boxes show first and third quartiles, the line inside the box indicates median value, and whiskers indicate 10–90 percentiles. Statistical results: asterisks indicate significant differences (*: *p* < 0.05; ns: non-significant). Coefficient *r* was 0.04 for serum and saliva BHB, and 0.59 for serum and saliva NEFAs (≥0.56: small effect; ≥0.64: moderate effect; ≥0.71: large effect).

The results are shown in Figure 7. No significant differences were observed in the BHB concentration in either the serum or the saliva. Regarding NEFAs, their concentration was significantly higher in animals with clinical symptoms in both the serum and the saliva.

## 4. Discussion

In this report, the measurement of BHB and NEFAs in bovine saliva using commercially available colorimetric methods has been validated. The two assays showed acceptable precision and accuracy. However, the presence of color in the sample due to food or debris significantly affected the measurement of NEFAs. The presence of feed has been previously described to affect the measurement of several biomarkers in bovine saliva [32]. As a consequence, the use of clean saliva would be preferable, discarding those samples with dirtiness or contamination.

The regression plots between paired serum–saliva samples indicated that the salivary BHB concentration was correlated with the serum, although the salivary values were 40-fold lower than in serum. This correlation did not exist in the case of NEFAs. In humans, it is known that NEFAs are secreted in the saliva [33], and their presence seems to play a role in the detection of fat from the diet [34] and therefore its concentration could be influenced by diet composition. Although this mechanism has not been proven in bovine species, the synthesis of NEFAs by the salivary glands and the possible influence of diet on the presence of NEFAs in saliva could be possible reasons explaining the lack of correlation between the serum and saliva NEFA concentrations as has been described in humans.

Once the measurement of the two biomarkers in the saliva was validated, their possible changes in cows with hyperketonemia and metritis were studied. Hyperketonemia is characterized by an energetic imbalance, and if it persists, the cows could experience clinical ketosis, primarily showing nonspecific signs such as losing weight or variations in milk production [35]. In addition, hyperketonemia increases the risk for infectious diseases, death, or culling [36,37,38]. Due to these reasons, balanced nutrition that provides a correct energetic balance is of high importance for farmers in order to prevent the disorder and avoid important economic losses. In addition, proper management at lactation and during dry periods is relevant to prevent this disorder. Once hyperketonemia is present, its early detection is of high importance in order to establish an appropriate treatment before the process worsens. The treatment is based on the administration of glucose or carbohydrate precursors such as propylene glycol or glycerol [39]. In addition, animals with metritis were considered for this study, since it is another prevalent disease at peripartum in which elevated NEFAs seems to have a role in pathogenesis [40,41].

In the cows with hyperketonemia, higher levels of BHB and NEFAs were found in the serum compared with cows without clinical signs and those with clinical signs of metritis. Hyperketonemia was considered with serum BHB levels > 1.2 mmol/L, a value which has been proposed as a cut-off for the diagnosis [42]. In addition, NEFA values > 0.76 mmol/L could be highly indicative of ketosis in cows [43], which was observed in 9 out of the 17 animals included in the hyperketonemic group. The saliva showed similar changes in BHB than in the serum, with significantly higher BHB concentrations in the cows with hyperketonemia (ranging 0.013–0.319 mmol/L) than in the animals with clinical signs of metritis or without clinical signs (ranging 0.005–0.0464 mmol/L). These results are in accordance with the linear relationship found between the salivary and the serum levels of BHB and would suggest the potential use of saliva as an analytical sample for BHB measurement as a biomarker of hyperketonemia. In contrast, the salivary NEFA concentrations were not increased in the cows with hyperketonemia compared to the other groups, probably due to a different dynamic of this analyte between the saliva and the serum. The ROC analyses showed the potential usefulness of the BHB measurement in saliva as a biomarker of hyperketonemia, whereas the salivary NEFA usefulness for this purpose was not observed.

Regarding the animals with clinical signs of metritis, the serum BHB and NEFA concentrations were not significantly different from the concentrations found in cows with no clinical signs, although the serum NEFAs showed a trend to be higher in cows with metritis (*p* = 0.056). An elevated NEFA concentration (>0.3 mmol/L) in the serum before calving has been found in cows that developed clinical signs of metritis within the first 20 days after calving [44]. In our report, the serum NEFA concentrations in the cows with clinical signs of metritis ranged 0.09–1.25 mmol/L, with 9 out of 18 animals showing > 0.3 mmol/L. In contrast, the salivary NEFA concentrations did not show significant differences compared with cows with no clinical signs. These results also support the idea that the salivary NEFA concentration does not reflect the circulating levels in serum.

The changes in the serum and saliva BHB and NEFA concentrations in the calves during the first days of life were also studied. In the case of BHB, no changes were observed during this time in serum or saliva. However, in our report, the median circulating NEFA levels found in the serum of newborn calves on the day of birth were 0.85 mmol/L, which are higher than those observed in healthy adult cows (0.14 mmol/L). This is in line with a previous report in which newborn calves had high blood values of NEFAs and decrease their values until they reach the second week of life, where the levels are similar to those expected in adult animals [14]. Different dynamics between the serum and the saliva were observed in the calves, since the highest values of NEFAs in the serum were found on day 0 and then decreased. In contrast, the highest values of salivary NEFAs were observed on days 1 and 2. Further studies should be undertaken to elucidate the reason of this difference. 

BRD is of great concern to veterinarians and producers, as the incidence has increased in recent years [45], causing major economic loss due to reduced weight gain, lower carcass quality, increased mortality of affected calves, and control measures applied to decrease the clinical impact [46]. The cause is multifactorial, in which several pathogens can be involved, such as BVDV, PI-3, bovine herpesvirus type 1, bovine coronavirus, and BRSV [47]. Viruses cause a first infection that immunosuppresses and damages the respiratory tract and the animals’ defense mechanisms. This leads to a secondary bacterial infection, in which *M. haemolytica*, *P. multocida*, *H. somni*, and *M. bovis* are commonly involved; most of them are opportunistic since they are usually found in the respiratory tract [48,49]. The diagnosis is challenging and usually based on clinical signs that include ocular and nasal discharge, cough, extended head, dry snout, floppy ears, poor coat, dull eyes, lethargy, social isolation, hypo-/anorexia, pyrexia, dehydration, tachypnea, and dyspnea [50], although with a low sensitivity and specificity [51]. The PCR tests were performed on the calves in our study to detect the presence of the common virus and bacteria related to BRD, but the animals were classified as asymptomatic or symptomatic according to the absence or presence of clinical signs at the time of sampling. Calves are managed based on the presence of symptomatology at feedlots, and the molecular results confirmed the presence of most of the pathogens described for this syndrome in both groups. The symptomatic animals showed elevated serum and salivary NEFAs when compared to the asymptomatic ones, probably due to lipid mobilization as a consequence of a reduced feed intake. However, no differences were seen in the BHB levels between the groups.

One limitation of this report is the low number of animals included in the different experimental trials. Although moderate and large size effects were observed for the significant results in the Kruskal–Wallis (adult cows’ experiment) and Friedman (newborn calves’ experiment) tests, which would be indicator of an appropriate statistical power, a low size effect was observed for the Mann–Whitney test (symptomatic and asymptomatic BRD calves’ experiment), indicating a limited power [52]. Therefore, it must be considered as a pilot study whose results should be tested with larger populations. In addition, the possible potential use of BHB in the saliva as a biomarker for the diagnosis, treatment, and monitoring of hyperketonemia should be assessed in these larger studies. Additionally, the reason for the divergences between NEFA concentration in the serum and the saliva should be explored as well as if NEFAs could be affected by elements and supplements in the diet [53]. Finally, the possible effect of stress on the biomarkers’ levels should also be evaluated.

## 5. Conclusions

BHB and NEFAs can be reliably measured in the saliva of bovine species using the commercially available automated spectrophotometric assays used in this study. The salivary BHB concentrations were significantly correlated with those present in the serum, whereas the salivary NEFA concentrations did not reflect those found in the serum in the conditions of this report. In the cows with hyperketonemia, salivary BHB was increased, allowing its possible use for detecting this pathologic condition. The NEFA concentration in saliva increased in the newborn calves after colostrum intake and also in the calves with symptomatic BRD compared with those without clinical symptoms. Further studies should be undertaken to elucidate the reason for these changes.

## Figures and Tables

**Table 1:** Intra-and inter-assay imprecisions obtained after measuring 2 samples with different analyte levels. Means and standard deviations (SDs) are expressed in mmol/L for both beta-hydroxybutyrate (BHB) and non-esterified fatty acids (NEFAs). The coefficients of variation (CVs) are expressed in %.

		Mean	SD	CV
BHB				
Intra-assay	High	0.0784	0.0021	2.71
	Low	0.0238	0.0016	6.55
Inter-assay	High	0.0749	0.0040	5.32
	Low	0.0228	0.0011	4.69
NEFAs				
Intra-assay	High	0.400	0.006	1.58
	Low	0.058	0.004	7.00
Inter-assay	High	0.408	0.023	5.67
	Low	0.066	0.009	13.55

## Data Availability

The raw data supporting the conclusions of this article will be made available by the authors on request.
